# Gene expression profiling as a potential predictor between normal and cancer samples in gastrointestinal carcinoma

**Published:** 2019-05-21

**Authors:** Panagiotis Apostolou, Aggelos C. Iliopoulos, Panagiotis Parsonidis, Ioannis Papasotiriou

**Affiliations:** ^1^ Research & Development Department, Research Genetic Cancer Centre S.A., Florina, Greece

**Keywords:** clustering analysis, gastrointestinal cancer, peripheral blood mononuclear cells, qRT-PCR

## Abstract

Analysis and comparison of gene expression profile among molecules, correlated with essential and crucial biological processes, is of primary importance in cancer research, since it provides significant info regarding the resistance to chemo/radiotherapy, risk for relapse or prediction of metastasis etc. In this study, gene expression profile is used for discriminating efficiently colon cancer cell lines from normal cells and cancer cells in blood samples of colon cancer patients and categorizing different types of gastrointestinal cancer. In particular, blood samples were collected from normal donors as well as from colon cancer patients. Peripheral blood mononuclear cells were isolated and gene expression analysis was performed for more than fifty genes. The same assays were performed for commercial cancer cell lines representing different types of gastrointestinal cancer. In order to examine whether the comparison of gene expression profile can lead to a thorough discrimination between cancer and normal states as well as between different cancer types, we performed clustering analysis based on hierarchical, and k-means algorithms. The clustering analysis efficiently separated: a) colon cancer cell lines from colon patients’ samples, b) normal from the colon cancer samples, c) gastric and pancreatic cancer from liver and colon types based. The exploitation of gene expression profile can be successfully used for the discrimination between normal vs cancer samples and/or for categorizing various types of cancer. This of course has important implications in cancer management since it enables the quick discrimination based on cells, isolated from bloodstream, needless of tissue examination or protocols requiring specialized equipment.

## INTRODUCTION

The gastrointestinal cancer includes not only parts of the gastrointestinal tract, but also different organs both from upper and lower digestive tract. Among them, colorectal cancer is the third leading cause of deaths, pancreatic cancer accounts approximately 6-7% of cancer deaths, concerning liver cancer, there was observed an increase in death rates the last decades [1]. The ability of early detection of the disease, in combination with improved therapeutic protocols, might contribute in better management of gastrointestinal cancer. The study of genes and proteins, involved in particular types of cancer, enable the physicians to predict the response to different therapeutic schemes, and/or progression of the disease.

The gene expression profile from tissue samples is widely used for classification, diagnosis, or prognosis of different diseases. Compared with conventional approach [2] and classification techniques, advantages are many-fold. Molecular biology assays enable the study of whole genome in a single experiment, while qRT-PCR technique is cost and time efficient. Furthermore, the accuracy of these methodologies has been increased, providing a powerful tool in medical community. In addition, the ability of exploring the genetic profile, through cells, which flow into the bloodstream, reduced the painful and not always easy procedure of biopsy. Liquid biopsies of circulating tumor cells have been validated and approved by the FDA as a useful prognostic method for various types of cancer [3]. The use of liquid biopsies for oncotherapy is still under investigation. In the present study gene expression levels of commercial cancer cell lines were tested, corresponding to different types of gastrointestinal cancer as well as cells from normal donors and patients suffering from colorectal cancer. The genes considered are well-studied genes involved in particular cellular processes, including cell cycle, apoptosis, metastasis, as well as other genes that are usually tested in cancer cases.

The above datasets were evaluated with clustering analysis, a significant technique in data mining process. This type of analysis is very useful in exploring hidden patterns and structure of the provided data. In general, cluster analysis aims on correctly assigning variables (in this case variables are for example the different types of cancer) to different groups. Ideally, this exploratory technique divides the variables into clusters so that variables within a cluster share similar biological features and roles [4]. In this study we used two commonly used clustering algorithms, namely hierarchical and *k*-means clustering, in order to examine whether the gene expression profiles derived from PBMCs can provide valuable information which can help towards the correct recognition-distinction between different types of cancer cells and between normal cells and cancer cells in blood samples of colon cancer patients.

## RESULTS

### Molecular assays

The qRT-PCR experiments did not reveal any repeatable expression pattern among the different samples that were studied. Between normal and cancer samples, the expression in the majority of genes was higher in cancer. Among the different gastrointestinal cancer cell lines, gastric cancer cell lines expressed higher genes correlated with metastasis and apoptosis, while there was not observed a clear difference between oncogenes, tumor suppressor genes, heat shock proteins or genes involved in growth factors pathways or cell cycle.

### Clustering

**Figure 1:**
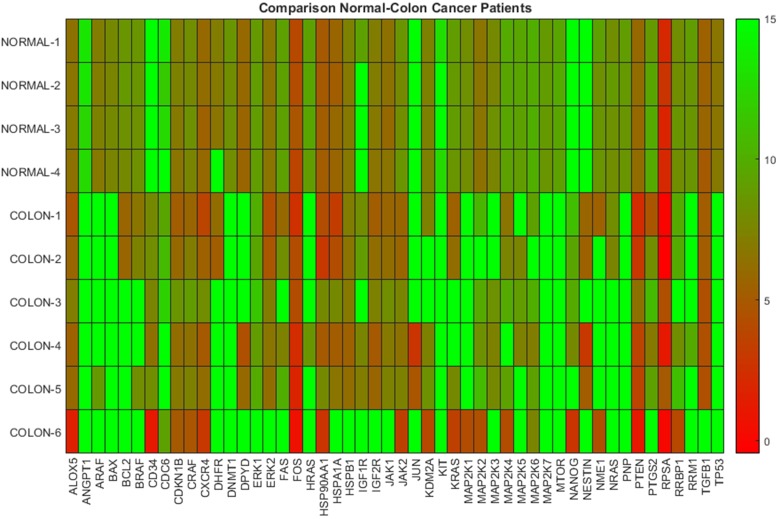
Gene expression data on PBMCs from normal donors and colon cancer patients. The heatmap presents DeltaCt values. The lower the DeltaCt the higher the gene expression. Therefore DeltaCt at 15 means that there the gene studied was not expressed. A visual distinction between the two groups is evident.

In Figure 1 the heat map diagram of the DeltaCt data for four normal subjects and six colon cancer patients based on PBMCs, is shown. This diagram exhibits the level of DeltaCt values of 50 genes (shown in x-axis) and represents it as a color. In particular, the lower the DeltaCt the higher the gene expression. Therefore, DeltaCt at 15 means that there is no expression. In the heat map maximum DeltaCt values are shown with open green and minimum with red. As it can be seen in the heat map, most colon cancer samples are associated with lower gene expression values (higher DeltaCts), while normal samples with higher-medium (lower DeltaCts) expression values. The visible inspection of the heat map indicates a possible distinction between the two groups. In Table 1, we provide results concerning the hierarchical and k-means clustering of the samples mentioned previously. The estimation of the indices of the groups was performed keeping fixed the number of clusters equal to two (which is the correct number of groups), since in this study we are concerned only with the successful assigning of indices in corresponding groups-samples. As it can be seen in Table 1, both clustering algorithms correctly distinguished the two distinct groups, namely normal and colon samples, indicating that gene expression profiles derived from PBMCs can be effectively used for the discrimination between normal and cancer types.

**Table 1:** Clustering of normal and colon samples based on gene expression profile based of PBMCs

GENE EXPRESSION PROFILE	HIERARCHICAL	*k*-MEANS
**Normal Sample (1)**	2	1
**Normal Sample (2)**	2	1
**Normal Sample (3)**	2	1
**Normal Sample (4)**	2	1
**Colon Cancer Patient Sample (1)**	1	2
**Colon Cancer Patient Sample (2)**	1	2
**Colon Cancer Patient Sample (3)**	1	2
**Colon Cancer Patient Sample (4)**	1	2
**Colon Cancer Patient Sample (5)**	1	2
**Colon Cancer Patient Sample (6)**	1	2

The indices of the clusters shown were estimated by two clustering methods, hierarchical and *k*-means.

**Figure 2:**
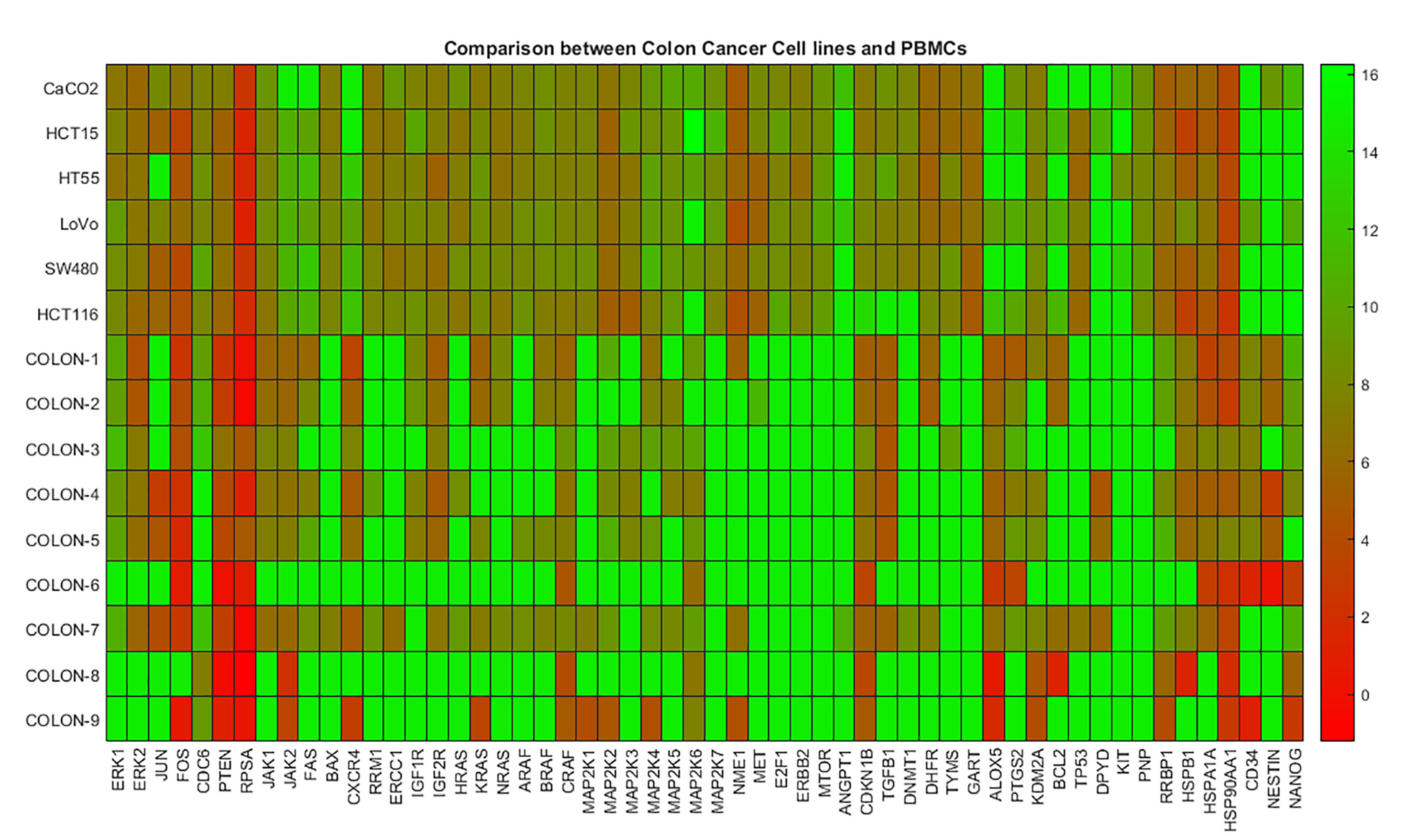
Gene expression data between colon cancer cell lines and colon patients PBMCs. The heatmap presents DeltaCt values. The lower the DeltaCt the higher the gene expression. Therefore DeltaCt at 15 means that there the gene studied was not expressed. A visual distinction between the two groups is evident.

In Figure 2 the heat map diagram of expression of 56 genes (shown in x-axis) corresponding to colon cancer as obtained from six commercial cancer cell lines and nine patient samples is shown. Similar to Figure 1, the open green color represents maximum DeltaCts values, while red minimum. As it is shown most cancer cell lines are related to lower gene expression values (higher DeltaCts), while PBMC samples with higher-medium expression values (lower DeltaCts). Therefore, the visible inspection of the heat map indicates a possible distinction between the two groups.

Table 2 presents results concerning the hierarchical and k-means clustering of gene expression profiles corresponding to the dataset used for generating the heat map shown in Figure 2. The maximum number of clusters was fixed equal to two. In particular, in this Table the indices of the clusters, as assigned to each cell line and sample, are shown. It can be seen that the indices are distributed correctly, indicating that the algorithms efficiently discriminated between the two categories present in the data, namely colon cancer cell lines from colon patients’ PBMCs.

**Table 2:** Clustering of gene expression profile derived from colon cancer cell lines and colon cancer patients’ samples

GENE EXPRESSION PROFILE COLON CANCER	HIERARCHICAL	*k*-MEANS
**Colon Cancer Cell Line (1)**	1	2
**Colon Cancer Cell Line (2)**	1	2
**Colon Cancer Cell Line (3)**	1	2
**Colon Cancer Cell Line (4)**	1	2
**Colon Cancer Cell Line (5)**	1	2
**Colon Cancer Cell Line (6)**	1	2
**Colon Cancer Patient Sample (1)**	2	1
**Colon Cancer Patient Sample (2)**	2	1
**Colon Cancer Patient Sample (3)**	2	1
**Colon Cancer Patient Sample (4)**	2	1
**Colon Cancer Patient Sample (5)**	2	1
**Colon Cancer Patient Sample (6)**	2	1
**Colon Cancer Patient Sample (7)**	2	1
**Colon Cancer Patient Sample (8)**	2	1
**Colon Cancer Patient Sample (9)**	2	1

The indices of the clusters shown were estimated by two clustering methods, hierarchical and *k*-means.

**Figure 3:**
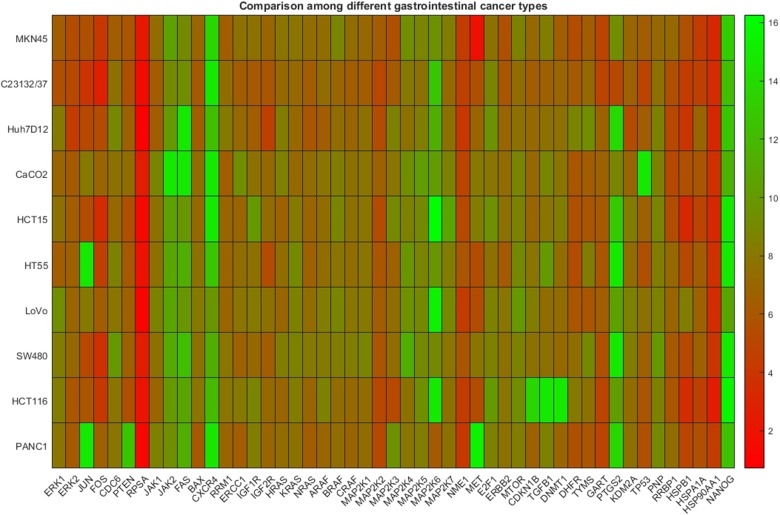
Gene expression data among cell lines representing different types of gastrointestinal cancer. The heatmap presents DeltaCt values. The lower the DeltaCt the higher the gene expression. Therefore DeltaCt at 15 means that there the gene studied was not expressed. The visual inspection does not reveal clustering patterns.

In Figure 3, the heat map plot concerning DeltaCts only for commercial cancer cell lines corresponding to four types of cancer is presented. In particular, 46 genes were considered (shown in x-axis), while the cancer types and the respective data include gastric (2 cell lines), liver (1 cell line), colon (6 cell lines), pancreatic (1 cell line). In this case, a visual inspection of the heat map, does not reveal a clear distinction between the cancer types mentioned, revealing a similar expression profile for all.

However, hierarchical and k-means clustering analysis revealed significant differences. These results are presented in Table 3 where the estimated indices of the groups are shown. In this case, the maximum number of clusters fixed equal to four. As it is shown, both algorithms managed to successfully distinguish the gastric and pancreatic cancer, from colon and liver cancer type. However, they failed to discriminate between the liver and colon types, while one colon cancer cell line sample was wrongly identified as a single cancer type-group.

**Table 3:** Clustering of gene expression profile derived from cancer cell lines for different types of cancer, namely gastric, liver, colon, pancreatic

Cancer Cell Lines	Hierarchical	*k*-means
**Gastric**	2	4
**Gastric**	2	4
**Liver**	3	3
**Colon**	3	3
**Colon**	3	3
**Colon**	3	3
**Colon**	3	3
**Colon**	1	1
**Colon**	3	3
**Pancreatic**	4	2

The indices of the clusters shown, were estimated by two clustering methods, hierarchical and *k*-means.

## DISCUSSION

In the present study we performed gene expression clustering analysis in PBMCs from colon cancer patients and non-cancer donors. PBMCs are peripheral cells with a nucleus and consisted of lymphocytes and monocytes. CTCs are a sub-population in PBMCs, and their study is important in cancer management. However, their detection and isolation requires different technologies, which are not always available in all laboratories. On the contrary, the gene expression protocols are quite easier and isolation of PBMCs is a routine in all the clinical centers. In addition the same panel was studied in commercial cancer cell lines representing different types of gastrointestinal cancer.

The genes that were used in this study are involved in different biological processes. In particular, the study of MAP kinases is essential, since these molecules contribute in proliferation, differentiation and cell cycle progression [5]. Genes like JUN and FOS participate in pathways controlling a lot of transcription factors. Different signal transduction pathways include oncogenes (RAS), tumor suppressor genes (PTEN, TP53), and many kinases (JAK, MAPK, MTOR, RAF) [6], [7], [8]. The contribution of growth factors in cancer is well known; therefore the study of genes incorporated in growth factors signals is also important in study of cancer samples (EGF, TGFB) [9], [10], [11]. In addition genes correlated with metastasis and/or apoptosis could be used to predict the progression of the disease (FAS, BAX, HGFR, BCL2, and NME1) [12], [13]. The group of genes we incorporated in our study was also enhanced with genes involved in metabolism (DHFR, TYMS, DPYD, DNMT1, GART, ALOX5, PTGS, KDM2, RRM1, CES, PNP) [14], [15], [16], cell cycle (CDC6, CDKN1B, CDKN2A) [17], repair (ERCC1), receptors (IGF1R, IGF2R) [18], chemokines (CXCL12, CXCR4) [19], translation (RPSA, RRBP1) [20] and heat shock proteins (HSPB1, HSPA1A, HSP90AA1) [21]. The study of all the above genes can reveal not only the behavior of cancer cells in drugs and other types of therapy, but also their metastatic abilities.

The expressions of the aforementioned genes was used as input data to clustering analysis methods in order to compare: a) colon cancer cell lines to colon cancer patients’ samples, b) normal samples to colon cancer samples and c) cell lines of different cancer types such as gastric, liver, colon and pancreatic. The clustering analysis was based on two commonly used algorithms, namely hierarchical and *k*-means clustering.

Indeed, the results of this study strongly confirm this hypothesis, since the algorithms efficiently managed to correctly discriminate: a) colon cancer cell lines from colon samples derived from patients, b) normal from the colon cancer samples, and c) gastric and pancreatic cancer from liver and colon types based on cell lines expression profiles. However, the algorithms falsely categorized liver cancer as colon, whereas predicted a colon cell line as a unique type of cancer. Concerning the categorization of a colon cell line as unique type, it has to be mentioned that the particular cell line (HCT-116) has a different cell type (epithelial-like), compared with the other colon cancer cell lines (epithelial), while is the only one which has monosomy after karyotyping [22]. The above might explain the different gene expression profile. Regarding the liver cancer cell line, there is not enough literature data about the metastatic profile of the patient [23]. There are plenty of cases where colon cancer metastasizes to liver, while it is rare the opposite. The common group of liver and colon cell lines might be explained if we had info about the donor of the cell line, since a metastasis could contribute in common gene expression profile [24], [25].

The results show that gene expression profile derived from PBMCs can provide significant information which can be used, to successfully discriminate between normal and patient subjects. Moreover, clear distinction between PMBCs and cancer cell lines for the same type of cancer (in this study colon cancer) was also achieved. Finally, the results from the application of clustering analysis to gene expression profiling derived cancer cell lines revealed efficient discrimination between different types of cancer. Even though the results seem promising, more experiments have to take place in order to obtain larger data sets, while the exploitation of more sophisticated clustering techniques is needed to verify and extend the results of this study. However, these issues will be addressed in following studies.

## MATERIALS AND METHODS

### Sample collection

40ml of blood was collected from nine patients suffering from colon cancer, while the same amount was collected from four healthy donors. Blood was placed in sterile 50 ml Falcon tubes (4440100, Orange Scientific, Braine-l’Alleud, Belgium) containing 7 ml of 0.02 M EDTA (E0511.0250, Duchefa Biochemie B.V., Haarlem, The Netherlands). Healthy individuals contained both male (3) and female (1) samples with age 32.5 ±6.65 years old, while cancer patients’ samples age was 62.66 ±6.48 years old. The patients’ samples included 5 males and 4 females. Regarding the stage of the disease, five of them were on stage IV, one in stage I, one in stage II and two with unknown stage. The study took place from January 2018 to March 2019.

### Cell lines

Cancer cell lines representing different types of gastrointestinal cancer (Table 4) were obtained from the European Collection of Cell Cultures (ECACC - HPA cultures, Salisbury, UK) and ATCC (Wesel, Germany). Cells were cultured in the appropriate medium, including heat-inactivated fetal bovine serum (10106-169, Invitrogen, NY, USA) and 2 mM L-glutamine (G5792, Sigma-Aldrich, Munich, Germany) at 37°C in a 5% CO_2_ atmosphere. Approximately 5,000,000 cells were isolated for further analysis. Cells were tested for contamination prior assays.

**Table 4:** Cell lines used in molecular assays

Cell Line	Type
**MKN45**	Gastric adenocarcinoma
**23132/37**	Gastric adenocarcinoma
**Huh 7D-12**	Hepatocellular carcinoma
**CaCO2**	Colon adenocarcinoma
**HCT-15**	Colon adenocarcinoma
**HT55**	Colon carcinoma
**LoVo**	Colon adenocarcinoma
**SW480**	Colon adenocarcinoma
**HCT-116**	Colon carcinoma
**PANC-1**	Caucasian pancreas

### Blood sample preparation

In order to separate blood cells, samples were centrifuged for 20 min at 2500 x g with 4 ml polysucrose solution (Biocoll separating solution 1077, Biochrom, Berlin, Germany). Then, the desired cell populations (mononuclear cells, lymphocytes, platelets and granulocytes) were collected, centrifuged and washed with phosphate-buffered saline (PBS) (P3813, Sigma-Aldrich). Cells were then placed in lysis buffer for 10 min to lyse the erythrocytes [(154 mM NH_4_Cl (31107, Sigma-Aldrich), 10 mM KHCO_3_ (4854, Merck, Darmstadt, Germany), and 0.1 mM EDTA in deionized water)]. At the end, samples were centrifuged and washed again.

### Microscopy evaluation

The isolated cells were evaluated microscopically. Cells were plated in slides and visualized in Primovert microscope (Zeiss), using ZEN software.

### Molecular analysis

RNA was isolated with RNeasy Mini Kit (74105, Qiagen, Hilden, Germany), and evaluated spectrophotometrically. The RNA was converted to cDNA with PrimeScript RT Reagent Kit (RR037A, Takara, Beijing, China). KAPA SYBR Fast Master Mix (2×) Universal (KK4618, KAPA Biosystems, MA, USA) was used for qPCR. The program included initial denaturation at 95°C for 2 min followed by 45 cycles of denaturation at 95°C for 10 s and annealing at 59°C for 30 s. A melting-curve program followed from 70°C to 90°C with 0.5°C increments for 5 s at each step. The primers for each specific gene, as well as for reference genes were designed in Beacon Designer 8 (Table 5). BLAST analysis was then followed to exclude primers that might amplify other genes. The primer pairs were evaluated for optimum Tm as well MgCl_2_ concentration. Gradient PCR (45^o^C-60^o^C) with different concentrations of MgCl_2_ were performed and then standard curve analysis was followed for each one using a reference RNA sample (740000-41; Agilent, CA, USA) and five different 10-fold serial dilutions. The qPCR products were evaluated both with agarose electrophoresis, as well as with melting curve analysis. Delta Ct (ΔCt: Ct of gene of interest minus Ct of housekeeping gene) value was used for analysis of experiments. Ct from threshold cycle, or the cycle of the reaction where the signal is detected.

**Table 5:** Primers used in PCR experiments

Gene	Forward Primer (5’–3’)	Reverse Primer (5’–3’)
**ACTB**	GCCCTGGACTTCGAGCAAGAGA	CAGGAAGGAAGGCTGGAAGAGTG
**ERK1**	CTGACGGAGTATGTGGCTACG	CCCAGGATGCCCAGAATGT
**ERK2**	CCAACCTGCTGCTCAACA	CTTGGTGTAGCCCTTGGAATT
**JUN**	GCCAAGAACTCGGACCTC	CGTTGCTGGACTGGATTATCA
**FOS**	GGCAAGGTGGAACAGTTATCTC	CCGCTTGGAGTGTATCAGTCA
**CDC6**	TTGGTGCTGATTGGTATTGCTAAT	TCTGGTATAAGGTGGGAAGTTCAA
**PTEN**	TAATTGCAGAGTTGCACAATATCCT	ACCAGTTCGTCCCTTTCCA
**RPSA**	TGAGAAGGCAGTGACCAAG	CGCTCCAGTCTTCAGTAGG
**JAK1**	CAGTGACACCATCATGTAAGGAG	ACAATATCTGGATTCTGCTCTTCAA
**JAK2**	ACTGAAGAGCACCTAAGAGACT	GATTACGCCGACCAGCAC
**FAS**	CCTCCTACCTCTGGTTCTTACG	ACAGTCTTCCTCAATTCCAATCC
**BAX**	CGCCGTGGACACAGACTC	CACAGGGCCTTGAGCACC
**CXCL12**	TGCCTCAGCGACGGGAAG	GCACAGTTTGGAGTGTTGAGAATT
**CXCR4**	TCAGTGAGGCAGATGACAGAT	GATGACAATACCAGGCAGGATAAG
**RRM1**	CGAGTGGAGACTAATCAGGACTG	TGCGGACACGACCTTGTT
**ERCC1**	CCTCCGCTACCACAACCT	CTGCTGGGGATCTTTCACATC
**IGF1R**	CCAATGCTTCAGTTCCTTCCA	CGCACAATGTAGTAACTCAGGTT
**IGF2R**	CGAGCAAGCGACCGAATG	GGCATCATCCTCACTCTCATCATA
**HRAS**	GTGCCTGTTGGACATCCTG	TCTGCTCCCTGTACTGGTG
**KRAS**	GTGGTAGTTGGAGCTGGTG	CCTGTAGGAATCCTCTATTGTTGG
**NRAS**	TGTGTGGTGATGTAACAAGATACT	TGAAGACAGCAACAGGAATACTT
**ARAF**	TCAAGTCTAACAACATCTTCCTACA	GCCATCCACAGCACAGAT
**BRAF**	GAGTCTTCCTGCCCAACAAA	GGTTTCTTCTCTCCATCCTGAATT
**CRAF**	GCACGCTTAGATTGGAATACTGA	TCCGAGCAAAGTTGTGTGTT
**MAP2K1**	AGGAGATGCGGCTGAGAC	TGGAGGAGGCTCGTTGAC
**MAP2K2**	AACTCCTGGACTATATTGTGAACGA	GGTGTGGTTTGTGAGCATCTT
**MAP2K3**	GGCACTATTCAGAGAGGGAGAC	CGCACGATAGACACAGCAATC
**MAP2K4**	CTAACACAAGTCGTGAAAGGAGAT	TTCGTAAGGCACAAGTTGACAA
**MAP2K5**	CCACAGTAATGGAACAGCAAGTA	GCCCGAGTATTCACCTTCAG
**MAP2K6**	ACCAGTTCCACACCACCTC	CACCTCGTCCCAGTTCCATTA
**MAP2K7**	ACAGTGGCGATTGTGAAGG	GCGTCTTGGCTTTGGAGT
**NME1**	GGGCTGAATGTGGTGAAGAC	ATGTATAATGTTCCTGCCAACTTGT
**MET**	CACTGCTTTAATAGGACACTTCTGA	AAGAGGACTTCGCTGAATTGAC
**E2F1**	CCACTGACTCTGCCACCATA	CAGCGGTTCTTGCTCCAG
**TERT**	GCAGGCGTACAGGTTTCAC	TTCAGGATGGAGTAGCAGAGG
**ERBB2**	CTCGGCTGCTGGACATTG	CCCACACAGTCACACCATAAC
**MTOR**	TGACAGTGGCTTCTAAGTCTACC	GCTCCTCGCTCACCATCAT
**ANGPT1**	CAGAGCAGCCTGATCTTACAC	CAAACCACCATCCTCCTGTTAA
**CES1**	GTCTCTGTTCTTGTTTTGTCTCCAT	ACCCAGCAGTGATAGCAATTTG
**CES2**	GGTGAACAGCAGCGTGTCC	CTGAGTCCTGGCCCTGGC
**CDKN1B**	CTGAGGACACGCATTTGGT	TTGAGTAGAAGAATCGTCGGTTG
**EGF**	ATGTAGCGGTTGTTCCTCAC	ATGGTTGTGGTCCTGAAGC
**TGFB1**	GAAACCCACAACGAAATCTATGAC	TTAACTTGAGCCTCAGCAGAC
**TGFB2**	GGCTTCACCATAAAGACAGGAA	ATATGTGGAGGTGCCATCAATAC
**TGFB3**	CGCTATATCGGTGGCAAGAATC	GACCTAAGTTGGACTCTCTTCTCA
**DNMT1**	ATCTCTTGAAGGTGGTGTTAATGG	GGTGCTGAAGCCGATGAG
**DHFR**	CAGCAGAGAACTCAAGGAACCT	GCCACCAACTATCCAGACCAT
**TYMS**	CAGATTATTCAGGACAGGGAGTTG	CATCAGAGGAAGATCTCTTGGATTC
**GART**	ATAATTGGCAGTGGAGGAAGG	TGATTGAGATGGCGGTATTTGA
**ALOX5**	CCTGTTCATCAACCGCTTCAT	TGACCCGCTCAGAAATAGTGT
**PTGS2**	AGCAGGCTAATACTGATAGGAGAG	AGGGTGTTAAATTCAGCAGCAATA
**KDM2A**	GGTATAAATGGTGCTGCGACAA	ACTGGCTGCCTCTTGATGA
**KDM2B**	CCAGCGATACGACGAGAAC	AGTTGAAATCTTTGCCCTCCAT
**BCL2**	CTGGAGAGTGCTGAAGATTGA	TCTACTTCCTCTGTGATGTTGTATT
**CDKN2A**	GTGGACCTGGCTGAGGAG	GGGATGTCTGAGGGACCTTC
**TP53**	GGAAGACTCCAGTGGTAATCTACT	GGCAGTGCTCGCTTAGTG
**DPYD**	AGGACGCAAGGAGGGTTT	AGCCAGGATACTCTCGATGTC
**KIT**	AACGAATGAGAATAAGCAGAATGAA	GCTTGGCAGGATCTCTAACA
**PNP**	ATGGAGAACGGATACACCTATGAA	CCTCCTAATCCAGAACCACAGA
**RRBP1**	GCCAAGGAGGAATCGGAGA	GGTGTAATTCTGTTGTGCCAAGA
**HSPB1**	CCTGGATGTCAACCACTTCG	GGGCAGCGTGTATTTCCG
**HSPA1A**	CTGGAGTCCTACGCCTTCA	CGAGATGACCTCTTGACACTTG
**HSP90AA1**	AGAGGCTGATAAGAACGACAAGT	TCTTCATCAATACCCAGACCAAGT
**CD34**	CCCATGCTGGAGGTGACATCTC	CCAGGGAGCCGAATGTGTAAAG
**NESTIN**	GAGACACCTGTGCCAGCCTTTCTTA	CTGGGCTCTGATCTCTGCATCTACAG
**NANOG**	CGTGTGAAGATGAGTGAAACTG	GGATGGGCATCATGGAAA
**18S rRNA**	TGCCCTATCAACTTTCGATGGTAGTC	TTGGATGTGGTAGCCGTTTCTCA
**GAPDH**	TGGAGAAGGCTGGGGCTCATT	GGTGCAGGAGGCATTGCTGATG

The primers are presented in 5’-3’ sequence.

All experiments were performed in triplicates, while both positive (Universal Human Reference RNA (740000-41, Agilent, CA, USA)) and negative controls.

### Clustering

The clustering analysis was based on two algorithms, hierarchical and *k*-means [26]. The calculations were performed using Software Matlab [27].

In the hierarchical clustering one tries to find the (dis)similarity between every pair of objects in the data. For this to take place, we need to calculate the distance between them. In this study, we used the Euclidean distance which is given by the following relation:

$$d_{st}^{2}=({\text{x}}_{s}-{\text{x}}_{t})({\text{x}}_{s}-{\text{x}}_{t})\text{'}$$ (1)

where *d*_st_ is the distance between the vector ***x***_*s*_ and ***x***_*t*_, corresponding to the data matrix. Continuously, based on the calculated distances which determine the proximity of the objects, we link the pairs of objects into binary clusters. The pairs are linked using the unweighted average distance function defined as:

$$d(r,s)=\frac{1}{n_{r}n_{s}}\sum_{i=1}^{n_{r}}\sum_{j=1}^{n_{s}}dist({\text{x}}_{rj},\;{\text{x}}_{sj})$$ (2)

where **r**, **s** are clusters (formed from other clusters), **n**_r_ and **n**_s_ are the number of objects in the clusters **r**, **s**, while **x**_ri_, **x**_sj_ are the *i*_th_ and *j*_th_ objects of clusters **r**, **s**.

On the other hand, *k*-means clustering [28], instead of operating on larger sets of (dis)similarity measures like hierarchical clustering, uses the actual observations to creating partitions on the data based on *k* mutually exclusive clusters. The clusters are formed based on the idea that objects within a cluster should be as much closer, while as far possible from the other objects-clusters. The main components in the clusters are their centers (or centroids) and the other member-objects. Mathematically, the center of a cluster is defined as minimum of the sum of the distances from all objects in the cluster. In this study, we used the squared Euclidean distance metric (Eq. 1) for the estimation of distances.

### Statistical analysis

The distribution of qPCR data was evaluated with the Kolmogorov–Smirnov test. The significant differences among various samples were calculated with one-way ANOVA.PAST 2.10 was used for the statistical analysis and significant *p* value was defined as <0.05.

## Abbreviations

PBMCs: Peripheral blood mononuclear cells
CTCs: Circulating tumor cells
